# Role of enteral nutrition in nonthyroidal illness syndrome: a retrospective observational study

**DOI:** 10.1186/s12902-015-0061-y

**Published:** 2015-11-04

**Authors:** Ranran Li, Jianan Ren, Qin Wu, Gefei Wang, Xiuwen Wu, Jun Chen, Guanwei Li, Zhiwu Hong, Huajian Ren, Yunzhao Zhao, Jieshou Li

**Affiliations:** Department of General Surgery, Jinling Hospital, Medical School of Nanjing University, 305 East Zhong Shan Road, Nanjing, 210002 China

**Keywords:** Nonthyroidal illness syndrome, Enterocutaneous fistula, Thyroid function, Enteral nutrition

## Abstract

**Background:**

The nonthyroidal illness syndrome (NTIS) is prevalent among patients with enterocutaneous fistula and is associated with poor outcomes. The present study aimed to explore the role of enteral nutrition (EN) therapy on thyroid function among patients with enterocutaneous fistula and NTIS.

**Methods:**

We conducted a retrospective observational study among patients with enterocutaneous fistula between January 2013 and April 2014. All enrolled patients received EN therapy. Thyroid function and other parameters were measured.

**Results:**

After administration of 4 weeks of EN therapy, NTIS was resolved in 66 patients (Group A), while it persisted in 14 patients (Group B). The overall treatment success rate was 82.50 %. There were no significant differences between groups A and B at baseline for all parameters, except for the time from admission to start of EN therapy. The logistic analysis revealed that the time from admission to start of EN therapy was a significant independent indicator for achieving resolution of NTIS in our cohort.

**Conclusions:**

This retrospective observational cohort study demonstrated that EN therapy can aid in the resolution of NTIS among patients with enterocutaneous fistula. These findings confirm the benefit of EN in the treatment of enterocutaneous fistula.

**Electronic supplementary material:**

The online version of this article (doi:10.1186/s12902-015-0061-y) contains supplementary material, which is available to authorized users.

## Background

Changes in the endocrine system that result from critical illnesses can cause multiple dysfunctions [1]. Also known as the euthyroid sick syndrome, the nonthyroidal illness syndrome (NTIS) commonly affects patients with enterocutaneous fistula [1, 2]. This syndrome is characterized by alterations in thyroid function, which are commonly reflected as low serum triiodothyronine (T3) and normal to low thyroxine (T4). Studies have suggested that low levels of thyroid hormones are predictors of poor outcome in sepsis and critical illness [3]. In our previous study, we reported an association between NTIS and poor outcome among patients with enterocutaneous fistula, indicating the clinical importance of these alterations [1].

To date, the pathogenesis of these endocrine alterations in NTIS is not fully understood. Previous studies have reported several approaches for improving thyroid function in different patient populations with NTIS [4–6]. Some studies suggested the association between nutritional deficiency and NTIS, indicating a potential role of enteral nutrition (EN) therapy in resolving NTIS [7, 8]. Conversely, other studies reported that underlying illness plays a key role in NTIS, suggesting that EN therapy would not be expected to aid in the resolution of the thyroid abnormalities [9, 10]. Because the prevalence of NTIS among patients with enterocutaneous fistula is increasing, we conducted a retrospective observational study to investigate the role of EN therapy in the resolution of NTIS.

## Methods

### Ethics statement

This retrospective, observational cohort study was conducted in accordance with the principles of good clinical practice and the Declaration of Helsinki. The study protocol was reviewed and approved by the Institutional Review Board at Jingling Hospital. Informed consent was not obtained as patient records and information were anonymized and de-identified prior to the study.

### Patients and study design

Based on a detailed medical chart review, we primarily enrolled consecutive patients with enterocutaneous fistula admitted between January 2013 and April 2014. The standard values of the variables assessed at our hospital are as follows: free triiodothyronine (FT3), 3.8–6.5 pmol/L; total triiodothyronine (TT3), 1.23–3.07 nmol/L; free thyroxin (FT4), 7.9–17.2 pmol/L; total thyroxin (TT4), 71–161 nmol/L; and thyroid stimulating hormone (TSH), 0.3–4.5 mU/L.

The criteria for NTIS applied in our study were as follows: (1) FT3 level less than 3.8 pmol/L and (2) TSH upper normal limit of 4.5 mU/L [2, 10]. In our department, all patients underwent thyroid homeostasis measurements upon admission for NTIS scanning, and a thyroid test every week to evaluate the thyroid function. Records of patients with NTIS at admission were primarily collected. The exclusion criteria were as follows (1) current use of EN, thyroid hormone and antithyroid drugs at admission; (2) history of coronary artery disease, myocardial infarction or cerebral infarction in the past month upon admission; (3) pregnancy or lactation; (4) a previous history of thyroidal, hypophyseal or hypothalamic disease; (5) age less than 18; (6) craniocerebral injury; (7) end-stage advanced malignant tumor; (8) medication history of thyroid hormone or antithyroid drugs; (9) and intracranial infection or hemorrhage in the past month. After excluding patients, clinical data of the remaining patients were collected to constitute our cohort. The observational period was limited to 4 weeks. The time point at admission was defined as week 0.

The primary outcome of our study was defined as the resolution of NTIS at week 4 after admission. We defined in this study that patients whose FT3 level is above 3.8 pmol/L at week 4 are those who recovered from NTIS. At the end of the study, patients who experienced resolution of NTIS in this cohort were assigned to Group A while others were assigned to Group B.

### EN therapy

In our department, the EN used for patients with enterocutaneous fistula was Peptisorb Liquid (Enteral Nutrition Suspension; Nutricia Company, Amsterdam, Holland). Once the output of intestinal fluid was limited (<200 ml/L) and patients were satisfactorily maintained on enteral feeding, EN was gradually introduced to reach full feeding. The EN therapy in our study was conducted as described in our previous study [11]. Briefly, EN was prescribed through a nasogastric or nasointestinal tube. The formula contained 1 cal/mL and had 500 kcal/bottle. The energy requirements were calculated using Long’s modified equation according to the usual body weight. No oral foods or fluids (except for water and weak tea) were allowed. The nutrition regimen during the study period remained almost unchanged.

### Data collection

For each enrolled patient, the following data were collected from the medical record: primary diseases, fistula location, underlying disease, Acute Physiology and Chronic Health Evaluation Score (APACHE II), time for initiation of enteral nutrition upon admission, white blood cell count (WBC), C-reactive protein (CRP), red blood cell count (RBC), platelet count (PCs), glutamic-pyruvic transaminase (GPT) and blood urine creatinine (Cr), FT3, TT3, FT4, TT4 and TSH. Baseline characteristics, including age and sex, were also collected.

In our department, venous blood for all laboratory tests was drawn between 5 am and 6 am. Serum indexes, including WBC, CRP, RBC, PCs, Cr, GPT FT3, TT3, FT4, TT4 and TSH were measured at least once a week to monitor the patients’ status. Laboratory values were calculated within 2 h after blood collection.

### Statistical analysis

Demographic data and laboratory parameters were summarized by frequency for categorical variables and means ± standard deviation (SD). The proportions were compared with chi-square test or Fisher’s exact test. Continuous variables were tested by means of the *t*-test with normal distribution or Wilcoxon rank-sum test with non-normal distribution. A logistic analysis was performed to assess the influence of each variable on treatment success rate. Survival analysis was conducted, and statistical analyses were performed with GraphPad Prism Software (version 5.01; GraphPad, San Diego, CA, USA) and SAS software (SAS 9.1.3; SAS Institute Inc., Cary, NC, USA). A P value <0.05 was considered statistically significant.

## Results

### Patient characteristics

**Fig. 1:**
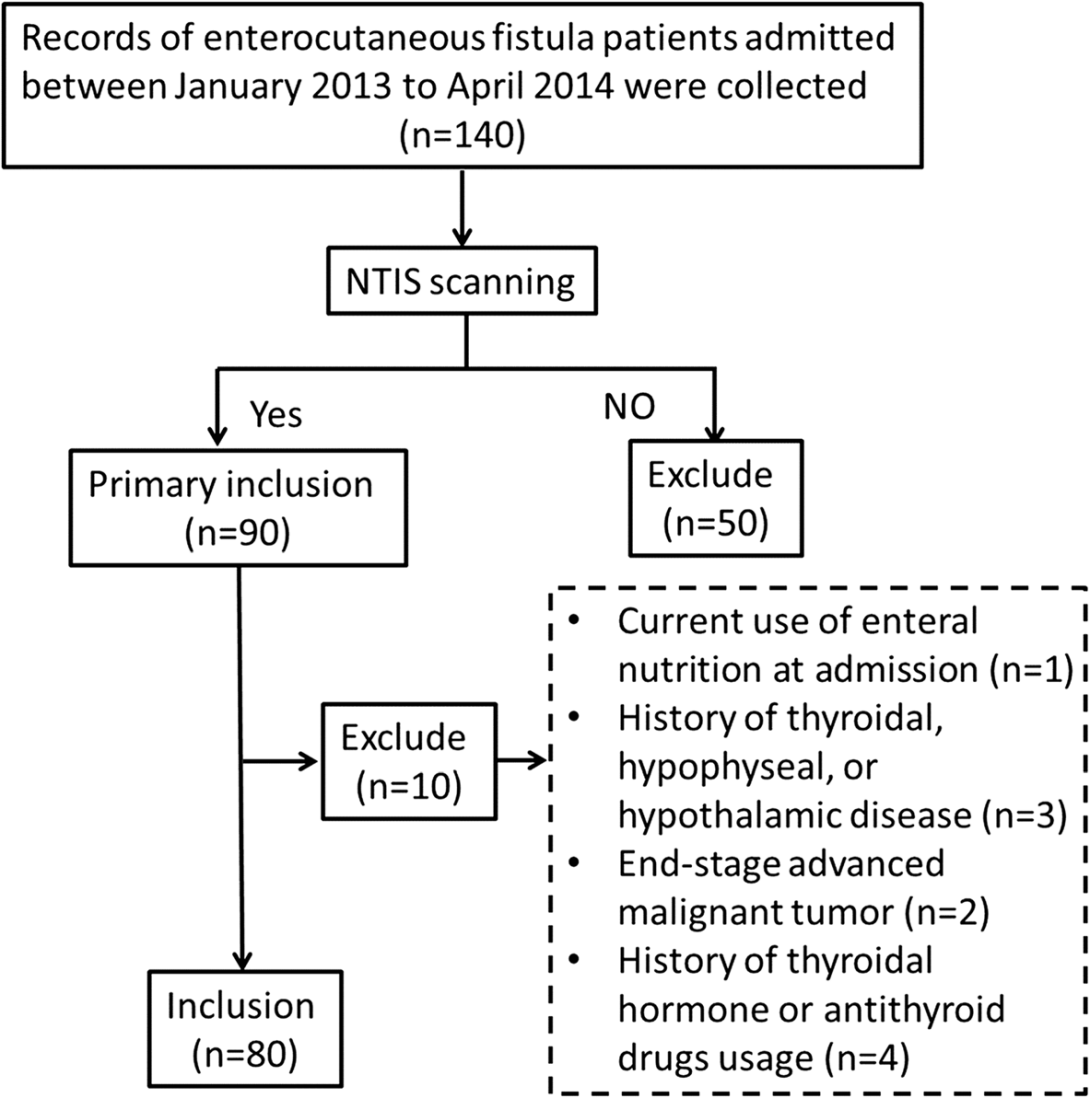
Flowchart of the current study. In total, 90 consecutive patients with NTIS were retrospectively enrolled from 140 patients with enterocutaneous fistula. Ten patients were excluded based on the exclusion criteria checking, and 80 patients were finally included

In total, 140 consecutive patients with enterocutaneous fistula were admitted to our department from January 2013 and April 2014. We retrospectively enrolled 90 consecutive patients with enterocutaneous fistula who met the NTIS criteria as primary inclusion. Ten patients met the exclusion criteria and were excluded from the study. Therefore, the final study cohort consisted of 80 patients (Fig. 1).

The general clinical characteristics of the study group at admission are presented in Table 1. Among the 80 cases, there were 58 males and 22 females (male-to-female ratio, 2.64:1). Patient had a mean (±SD) age of 48.05 ± 13.43 years. The most common causes for enterocutaneous fistula development in our study were trauma and surgical complications. The most common location of enterocutaneous fistula was the small bowel. A total of 24 patients had a positive history of diabetes mellitus, and 12 had chronic obstructive pulmonary diseases. The time from admission to start of EN therapy varied during our observational period. During the EN treatment, 8 patients (9.76 %) had diarrhea caused by the EN infusion, and symptoms were managed as stated in the medical records. None of the patients discontinued EN because of poor tolerance to EN therapy during the study period. No mortality occurred among the cohorts.

**Table 1:** General clinical condition of the study group at admission

Parameters	All subjects (*N* = 80)
Gender (Male, n %)	58 (72.50 %)
Age (yrs, mean ± SD)	48.05 ± 13.43
Severity scores, mean ± SD	
APACHE II score	16.27 ± 2.05
Primary diagnosis (n %)	
Trauma/Surgery complication	50 (62.50 %)
IBD	16 (20.00 %)
Pancreatitis	6 (7.50 %)
Others	8 (10.00 %)
Fistula location	
Duodenum	12 (15.00 %)
Colon	16 (20.00 %)
Small bowel	32 (40.00 %)
Multiple viscera^a^	20 (25.00 %)
Underlying disease, n ( %)	
Untreated cancer	8 (10.00 %)
DM	24 (30.00 %)
COPD	12 (15.00 %)
None	36 (45.00 %)
Time for initiation of enteral nutrition, n ( %)	
< 7 day	20 (25.00 %)
7–14 days	30 (37.50 %)
15–21 days	22 (27.50 %)
> 22 days	8 (10.00 %)

*IBD* inflammatory bowel diseases, *DM* diabetes mellitus, *COPD* chronic obstructive pulmonary diseases, *LOS* length of stay, *IQR* interquartile range

^a^Multiple viscera includes small bowel/colon, duodenum/small bowel or pancreas/duodenum/small bowel

### Changes in thyroid function

**Fig. 2:**
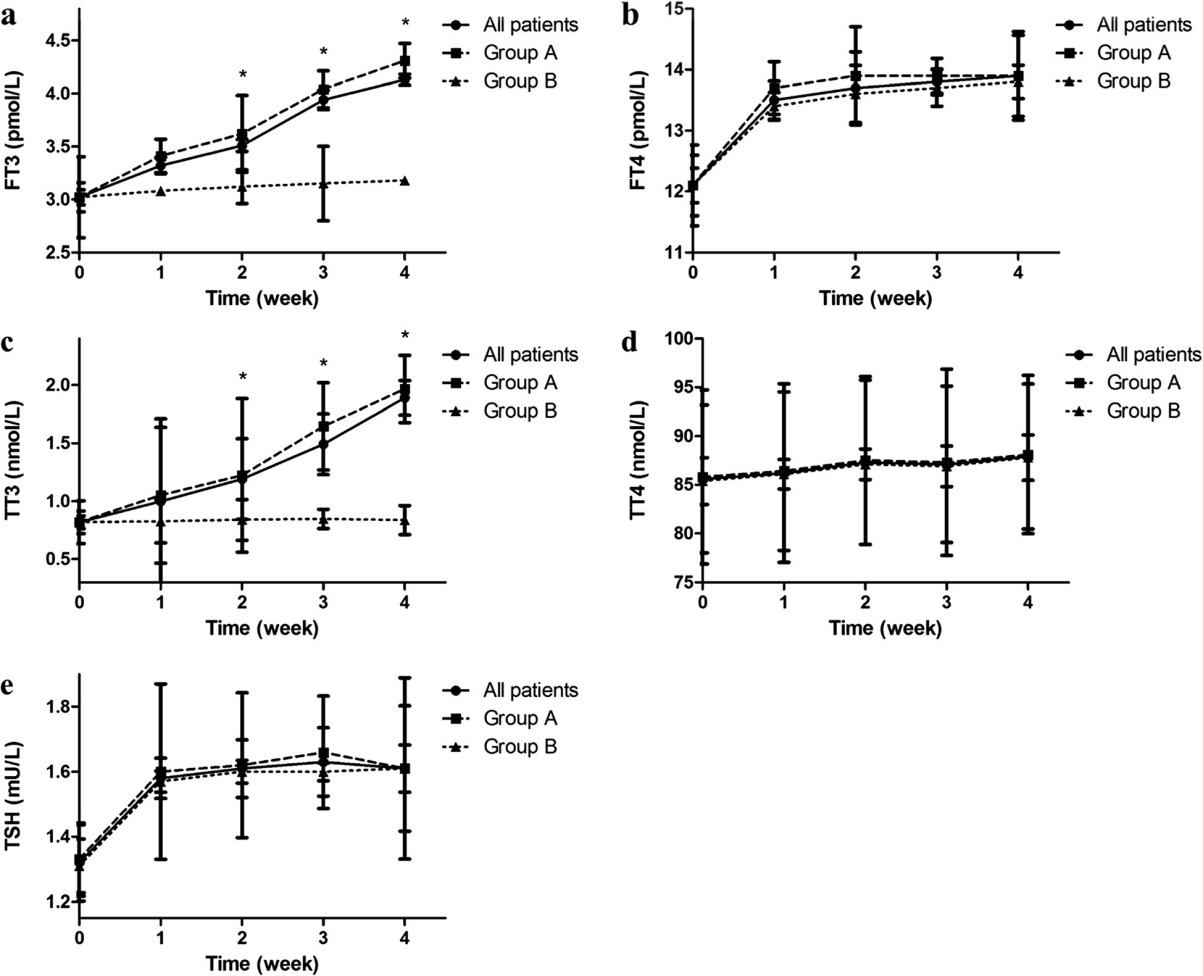
Changes of thyroidal function during the observational period. Serum (**a**) FT3 and (**c**) TT3 kept significantly increasing in group A compared with group B while other index [(**b**) FT4, (**d**) TT4 and (**e**) TSH] remained stable. (* indicated a significant difference between group A and group B)

Figure 2 and Additional file 1: Tables S1 shows the changes of thyroid function in this cohort during the observational period. During our observational period, serum FT3 concentrations were significantly increased, from 3.02 ± 0.138 at the baseline to 4.13 ± 0.052 in the last week (*p* <0.001), while serum level of FT4, TT4 and TSH kept stable during our observation.

According to our definition, 66 patients experienced resolution of NTIS (Group A) while 14 patients did not (Group B). The overall treatment success rate for both groups was 82.50 % (66/80). Table 2 presents the general clinical condition of both groups. The time from admission to start for EN therapy was significantly different between Groups A and B. Thyroidal function changes between those two groups in our study were displayed in Fig. 2 and Additional file 2: Table S2. Serum FT3 and TT3 level increased significantly faster in group A compared with those in group B. At the end of the second week after admission, serum FT3 and TT3 in group A were significantly higher than those in group B.

**Table 2:** General clinical condition of the group A and group B at admission

Parameters	Group A (*N* = 66)	Group B (*N* = 14)	*P*
Gender (Male, n %)	48 (72.72 %)	10 (71.43 %)	0.944
Age (yrs, mean ± SD)	47.69 ± 13.03	48.71 ± 15.17	0.708
Severity scores, mean ± SD			
APACHE II score	15.64 ± 1.29	16.47 ± 2.87	0.248
Primary diagnosis (n %)			0.679
Trauma/Surgery complication	44 (66.67 %)	6 (42.86 %)	
IBD	12 (18.18 %)	4 (28.57 %)	
Pancreatitis	4 (6.06 %)	2 (14.29 %)	
Others	6 (9.09 %)	2 (14.29 %)	
Fistula Location			0.139
Duodenum	10 (15.15 %)	2 (14.29 %)	
Colon	16 (24.24 %)	0 (0.00 %)	
Small bowel	28 (42.42 %)	4 (28.57 %)	
Multiple viscera^a^	12 (18.18 %)	8 (57.14 %)	
Underlying disease, n (%)			0.634
Untreated cancer	6 (9.09 %)	2 (14.29 %)	
DM	20 (30.30 %)	4 (28.57 %)	
COPD	12 (18.18 %)	0 (0.00 %)	
None	28 (42.42 %)	8 (57.14 %)	
Time for initiation of enteral nutrition, n (%)			0.013
< 7 day	18 (27.27 %)	2 (14.29 %)	
7–14 days	28 (42.42 %)	2 (14.29 %)	
15–21 days	18 (27.27 %)	4 (28.57 %)	
>22 days	2 (3.03 %)	6 (42.86 %)	
Hospital cost, median (IQR), dollar	28,502.82 (31,494)	32,042.04 (28,492)	0.003
WBC, ×10^9^/L, mean ± SD	10.30 ± 6.31	10.54 ± 1.47	0.123
CRP, mg/L, mean ± SD	73.53 ± 62.11	71.82 ± 25.11	0.223
RBC, ×10^9^/L, mean ± SD	3.21 ± 0.73	3.16 ± 0.54	0.705
GPT, U/L, mean ± SD	46.62 ± 25.36	44.27 ± 17.96	0.512
PCs, ×10^9^/L, mean ± SD	126.48 ± 82.38	122.66 ± 78.53	0.189
Cr, umol/L, mean ± SD	56.28 ± 49.98	53.50 ± 34.59	0.661

*P* value was calculated between group A and group B

*IBD* inflammatory bowel diseases, *DM* diabetes mellitus, *COPD* chronic obstructive pulmonary diseases, *LOS* length of stay, *IQR* interquartile range, *WBC* white blood cell counts, *CRP* C-reactive protein, *RBC* red blood cell counts, *PCs* platelet counts, *GPT* glutamic-pyruvic transaminase, *Cr* blood urine creatinine

^a^Multiple viscera includes small bowel/colon, duodenum/small bowel or pancreas/duodenum/small bowel

The logistic regression were performed with variables that associated with treatment success, respectively, in our study, including age, gender, primary diagnosis, fistula location, underlying disease, APACHEIIscore and time for initiation of enteral nutrition, to demonstrate their association with treatment success. Table 3 revealed that initial time of EN was a significant independent indicator with treatment success in our cohort.

**Table 3:** Multivariate analysis of time for initiation of enteral nutrition and other covariates associated with NTIS recovery

Variables	Hazard ratio	95 % CI		*P* value
		lower	upper	
Age				
> 48	20.829	0.639	679.079	0.088
= <48	1.000	–	–	–
Gender				
Male	1.000	–	–	–
Female	0.019	0.001	764.165	0.115
Primary diagnosis				
Non- surgical complication	10.030	0.149	674.056	0.283
Surgical complication	1.000	–	–	–
Fistula Location				
Single	1.000	–	–	–
Multiple	0.037	0.001	1.093	0.058
Underlying disease				
Yes	1.000			
No	1.405	0.104	18.898	0.798
APACHE II				
> 16	1.000			
= <16	1.461	0.869	2.456	0.152
FT3 at the baseline				
> 3.02	1.000	–	–	–
= <3.02	0.213	0.009	5.272	0.345
Time for initiation of enteral nutrition				
= <2 week	28.204	1.041	764.165	0.047
> 2 week	1.000	–	–	–

*FT* free thyroxin, *95 % CI* 95 % confidence interval

### Status of other serum indices

Table 4 shows the changes of other serum indices measured in our study. For all patient populations, all the laboratory tests presented no significant differences over time, except for some isolated indices. CRP kept decreasing over time in our study and reached a significantly low level at the end of our observational period. The same trend was observed for GPT. PCs in all populations kept increasing to a relatively normal level. In subgroup analysis, changes of serum indices measured in our study shared a similar trend between groups, without any significant differences between Groups A and B.

**Table 4:** Laboratory tests of all enrolled patients after admission

Time	Parameters	All subjects (*N* = 80)	Group A (*N* = 66)	Group B (*N* = 14)	*P*-value^a^	*P*-value^b^
Baseline	WBC, ×10^9^/L, mean ± SD	10.35 ± 6.52	10.30 ± 6.31	10.54 ± 1.47	0.785	–
	CRP, mg/L, mean ± SD	72.03 ± 54.21	73.53 ± 62.11	71.82 ± 25.11	0.496	–
	RBC, ×10^9^/L, mean ± SD	3.17 ± 0.68	3.21 ± 0.73	3.16 ± 0.54	0.389	–
	GPT, U/L, mean ± SD	45.21 ± 28.03	46.62 ± 25.36	44.27 ± 17.96	0.443	–
	PCs, ×10^9^/L, mean ± SD	124.67 ± 62.42	126.48 ± 82.38	122.66 ± 78.53	0.453	–
	Cr, umol/L, mean ± SD	54.78 ± 54.11	56.28 ± 49.98	53.50 ± 34.59	0.557	–
Week 1	WBC, ×10^9^/L, mean ± SD	9.87 ± 5.17	9.89 ± 5.34	9.35 ± 3.47	0.745	0.466
	CRP, mg/L, mean ± SD	42.58 ± 27.47	43.61 ± 28.18	40.91 ± 11.85	0.058	0.022
	RBC, ×10^9^/L, mean ± SD	3.19 ± 0.72	3.20 ± 0.78	3.18 ± 0.17	0.177	0.597
	GPT, U/L, mean ± SD	36.01 ± 22.28	37.82 ± 13.51	35.29 ± 10.83	0.581	0.180
	PCs, ×10^9^/L, mean ± SD	141.81 ± 57.02	143.69 ± 52.76	140.11 ± 12.54	0.184	0.388
	Cr, umol/L, mean ± SD	52.47 ± 43.73	54.20 ± 19.97	51.86 ± 7.09	0.588	0.806
Week 2	WBC, ×10^9^/L, mean ± SD	10.01 ± 6.13	10.12 ± 7.22	9.89 ± 2.25	0.685	0.631
	CRP, mg/L, mean ± SD	28.62 ± 37.38	30.88 ± 23.48	26.69 ± 20.87	0.047	0.017
	RBC, ×10^9^/L, mean ± SD	3.21 ± 0.71	3.22 ± 0.23	3.19 ± 0.50	0.472	0. 614
	GPT, U/L, mean ± SD	29.12 ± 12.38	30.24 ± 16.57	28.59 ± 14.87	0.841	0.158
	PCs, ×10^9^/L, mean ± SD	153.32 ± 37.32	155.54 ± 56.77	151.08 ± 39.52	0.687	0.069
	Cr, umol/L, mean ± SD	43.55 ± 27.31	45.13 ± 16.47	42.32 ± 37.54	0.343	0.635
Week 3	WBC, ×10^9^/L, mean ± SD	9.92 ± 4.38	9.96 ± 3.82	9.84 ± 2.87	0.776	0.508
	CRP, mg/L, mean ± SD	18.52 ± 10.41	19.13 ± 13.13	18.23 ± 9.33	0.214	0.012
	RBC, ×10^9^/L, mean ± SD	3.20 ± 0.85	3.22 ± 0.93	3.19 ± 0.12	0.462	0.607
	GPT, U/L, mean ± SD	21.61 ± 9.12	22.47 ± 12.92	20.38 ± 7.23	0.596	0.031
	PCs, ×10^9^/L, mean ± SD	176.66 ± 50.56	178.89 ± 67.53	175.12 ± 24.68	0.295	0.045
	Cr, umol/L, mean ± SD	37.93 ± 15.09	39.55 ± 10.89	35.23 ± 27.42	0.493	0.431
Week 4	WBC, ×10^9^/L, mean ± SD	9.16 ± 5.23	9.20 ± 7.89	9.12 ± 3.69	0.823	0.383
	CRP, mg/L, mean ± SD	14.11 ± 6.17	14.55 ± 7.95	19.39 ± 3.98	0.069	0.005
	RBC, ×10^9^/L, mean ± SD	3.22 ± 0.44	3.25 ± 0.59	3.20 ± 0.34	0.406	0.621
	GPT, U/L, mean ± SD	18.73 ± 9.91	19.72 ± 8.26	17.87 ± 7.40	0.622	0.010
	PCs, ×10^9^/L, mean ± SD	187.53 ± 15.93	189.37 ± 26.71	185.72 ± 42.46	0.641	0.033
	Cr, umol/L, mean ± SD	35.64 ± 28.54	37.87 ± 27.95	33.34 ± 18.92	0.838	0.385

*WBC* white blood cell counts, *CRP* C-reactive protein, *RBC* red blood cell counts, *PCs* platelet counts, *GPT* glutamic-pyruvic transaminase, *Cr* blood urine creatinine

^a^ *P* value is compared between group A and group B

^b^ *P* value is compared between the index at varied time point and the same index at the baseline among all subjects

## Discussion

In the current study, we retrospectively enrolled patients with NTIS admitted at our center and investigated the role of EN therapy in the treatment of NTIS. We observed that 66 out of 80 patients experienced resolution of NTIS after EN therapy. Further, we compared the clinical information between groups in terms of efficacy. A comparison between Groups A and B indicated that the time from admission to start of EN therapy was significantly different. Results from logistic regression analysis revealed that the time from admission to start of EN was a significant independent indicator of NTIS outcome. To our knowledge, this is the first study to focus on the potential role of EN in patients with NTIS and enterocutaneous fistula.

The term NTIS is used to describe the deranged TH profile observed in nonthyroidal illnesses that is characterized mainly by decreased serum T3 and⁄or T4 and in some cases suppressed TSH levels [12]. NTIS is a common alteration in thyroid function observed in about 70 % of hospitalized patients, with or without acute systemic illnesses [13]. In this study, the prevalence of NTIS among patients with enterocutaneous fistula was 64.28 % (90/140), which accorded with our previous study, showing the prevalence of this alteration [1].

It was proven that thyroid function abnormalities can occur within hours of acute illness, and the magnitude of these alterations correlates with the severity of the disease. Additionally, the lowest T3 and T4 values are associated with decreased survival [2]. Several studies have reported the association between NTIS and poor outcome [14, 15]. Our previous study also showed that patients with enterocutaneous fistula and NTIS presented worse clinical outcome and prognosis [1].

The etiology of the NTIS has been demonstrated to be multifactorial. It has been suggested that increased levels of endogenous or exogenous glucocorticoids, cytokines and catecholamines are implicated in the dysregulation of thyroid hormones [16–18]. The increased levels of these substances, occurring in conjunction with critical illness and severely hypocaloric diets, favor the conversion of T4 [19, 20]. Changes in thyroid function are commonly seen as adaptive changes in times of stress. However, consideration has also been given to the possibility that patients who have NTIS may not respond to elevated TSH because of central hypothyroidism secondary to systemic illness [2].

Several studies have examined the efficacy of treating NTIS with thyroxine administration, but the results were inconclusive and controversial [21]. The recovery of thyroid function after administration of EN in patients with NTIS indicates the potential role of EN in reversing NTIS [22]. In this study, we observed an improvement in thyroid function in a cohort of patients with enterocutaneous fistula after EN therapy. A total of 82.50 % (66/80) patients experienced resolution of NTIS, indicating a beneficial role of EN in NTIS.

We further compared the general clinical condition between Groups A and B to investigate the differences between patients who recovered from NTIS after EN therapy and those who did not. Based on that comparison, we found that the difference between groups was the time from admission to start of EN therapy. In the logistic analysis, we chose 2 week as the threshold because the median initial time of EN therapy in our patient cohorts is closed to that. We found that patients who received EN therapy within 2 weeks after 2 weeks have a significantly higher chance to recovery from NTIS.

The improvement in thyroid function that we observed after EN therapy in this study may be ascribed to multiple factors. EN therapy may have a direct impact on thyroid function or it may exert an indirect effect by influencing the prognosis of the primary disease. Because the changes of the underlying disease during the treatment were not significantly different between both groups (shown in Table 4), we speculate that EN exerts a direct effect on the improvement of the thyroid function that led to the resolution of NTIS, rather than on underlying diseases among enterocutaneous fistula patients. From this study, we can also conclude that additional thyroxine is not needed in patients under EN therapy with NTIS. Still, a well-designed randomized clinical study is needed to draw a definitive conclusion.

The observational period in our study was limited to 4 weeks because the average hospital stay for patients with enterocutaneous fistula who were admitted to our department was around 4 weeks. Usually, patients with enterocutaneous fistula would be admitted to our department to control infection and maintain homeostasis. When the clinical condition of patients was stable, they would be transferred to another ward. Once a stable condition and homeostasis were achieved, they would return to our department for definitive surgery. Measurement of thyroid function parameters was not available for our cohort after hospital discharge. Thus, this study was limited to 4 weeks of hospitalization.

The present study has several limitations. First, this was just a retrospective observational cohort study from a single medical center. Seasonably, data from different regional hospitals might increase the external validity of our conclusions. Second, as a retrospective study, the validity of our conclusions might increase if the sample size was expanded. In our study, we did not include indices that affected the nutritional status because of the short observational period. We excluded patients with varied comorbidities which limited our patient cohorts. Finally, well-organized randomized, double-blind clinical trials and systematic analysis of the role of EN in enterocutaneous fistula patients with NTIS are needed.

## Conclusions

In conclusion, this retrospective observational cohort study demonstrated that EN therapy can aid resolution of NTIS in enterocutaneous fistula patients. These findings confirm the benefit of EN in the treatment of enterocutaneous fistula. Further studies are expected to investigate the underlying mechanisms of this effect in the future.

## Additional files

Additional file 1: Table S1. Changes of thyroid function of the whole patient cohort after admission. (DOCX 22 kb) Additional file 2: Table S2. Changes of thyroid function after admission. (DOCX 25 kb)

## Abbreviations

APACHE II: Acute Physiology and Chronic Health Evaluation Score
Cr: blood urine creatinine
CRP: C - reactive protein
EN: enteral nutrition
FT3: free triiodothyronine
FT4: free thyroxin
GPT: glutamic-pyruvic transaminase
NTIS: the nonthyroidal illness syndrome
PCs: platelet count
RBC: red blood cell count
SD: standard deviation
T3: serum triiodothyronine
T4: thyroxine
TSH: thyroid stimulating hormone
TT3: total triiodothyronine
TT4: total thyroxin
WBC: white blood cell count
