# Supplementary material for: Role of enteral nutrition in nonthyroidal illness syndrome: a retrospective observational study
Source: BMC Endocr Disord. 2015 Nov 4;15:69. doi: 10.1186/s12902-015-0061-y (PMC4632465; doi:10.1186/s12902-015-0061-y)
Supplement: Additional file 1: Table S1. — Changes of thyroid function of the whole patient cohort after admission. (DOCX 22 kb) [file 12902_2015_61_MOESM1_ESM.docx]

**Additional file 1: Table S1. Changes of thyroid function of the whole patient cohort after admission**

| **Parameters** | **Week 0 (Baseline)** | **Week 1** | **Week 2** | **Week 3** | **Week 4** |
| --- | --- | --- | --- | --- | --- |
| Numbers, n | 80 | 80 | 80 | 78 | 75 |
| FT3, pmol/L, mean±SD | 3.02±0.138 | 3.32±0.078 | 3.51±0.056^*^ | 3.94±0.091^*^ | 4.13±0.052^*^ |
| FT4, pmol/L, mean±SD | 12.1±0.286 | 13.5±0.311 | 13.7±0.591 | 13.8±0.212 | 13.9±0.664 |
| TT3, nmol/L, mean±SD | 0.817±0.056 | 0.998±0.712 | 1.19±0.348^*^ | 1.49±0.262^*^ | 1.89±0.149^*^ |
| TT4, nmol/L, mean±SD | 85.6±7.58 | 86.2±9.17 | 87.3±8.43 | 87.1±8.02 | 87.9±7.44 |
| TSH, mU/L, mean±SD | 1.32±0.117 | 1.58±0.062 | 1.61±0.089 | 1.63±0.105 | 1.61±0.193 |

FT: free thyroxin; TT: total thyroxin; TSH: thyroid stimulating hormone;

* P value is less than 0.05 compared with the baseline within the group;
