# Supplementary material for: Role of enteral nutrition in nonthyroidal illness syndrome: a retrospective observational study
Source: BMC Endocr Disord. 2015 Nov 4;15:69. doi: 10.1186/s12902-015-0061-y (PMC4632465; doi:10.1186/s12902-015-0061-y)
Supplement: Additional file 2: Table S2. — Changes of thyroid function after admission. (DOCX 25 kb) [file 12902_2015_61_MOESM2_ESM.docx]

**Additional file 2: Table S2. Changes of thyroid function after admission**

| **Group** | **Parameters** | **Week 0 (Baseline)** | **Week 1** | **Week 2** | **Week 3** | **Week 4** |
| --- | --- | --- | --- | --- | --- | --- |
|  | Numbers, n | 66 | 66 | 66 | 64 | 61 |
| Group A | FT3, pmol/L, mean±SD | 3.02±0.383 | 3.41±0.158 | 3.62±0.363^*△^ | 4.04±0.175^*△^ | 4.31±0.164^*△^ |
|  | FT4, pmol/L, mean±SD | 12.1±0.666 | 13.7±0.433 | 13.9±0.804 | 13.9±0.285 | 13.9±0.726 |
|  | TT3, nmol/L, mean±SD | 0.817±0.186 | 1.050±0.587 | 1.221±0.662^*△^ | 1.644±0.375^*△^ | 1. 966±0.289^*△^ |
|  | TT4, nmol/L, mean±SD | 85.8±8.94 | 86.4±8.13 | 87.5±8.62 | 87.3±9.56 | 88.1±8.12 |
|  | TSH, mU/L, mean±SD | 1.33±0.112 | 1.60±0.270 | 1.62±0.223 | 1.66±0.173 | 1.61±0.279 |
|  | Numbers, n | 14 | 14 | 14 | 14 | 14 |
| Group B | FT3, pmol/L, mean±SD | 3.02±0.072 | 3.08±0.035 | 3.12±0.159 | 3.15±0.352 | 3.18±0.033 |
|  | FT4, pmol/L, mean±SD | 12.1±0. 497 | 13.4±0.229 | 13.6±0.469 | 13.7±0.299 | 13.8±0.273 |
|  | TT3, nmol/L, mean±SD | 0.817±0.098 | 0.825±0.187 | 0.838±0.176 | 0.846±0.083 | 0.853±0.125 |
|  | TT4, nmol/L, mean±SD | 85.4±2.41 | 86.1±1.52 | 87.1±1.57 | 86.9±2.08 | 87.8±2.33 |
|  | TSH, mU/L, mean±SD | 1.31±0.083 | 1.57±0.033 | 1.60±0.035 | 1.60±0.027 | 1.61±0.073 |

FT: free thyroxin; TT: total thyroxin; TSH: thyroid stimulating hormone;

* P value is less than 0.05 compared with the baseline within the group;

△ P value is less than 0.05 compared with Group B at the same time point;
